# Iron and zinc content of selected foods in the diet of schoolchildren in Kumi district, east of Uganda: a cross-sectional study

**DOI:** 10.1186/1475-2891-10-81

**Published:** 2011-08-09

**Authors:** Ida Tidemann-Andersen, Hedwig Acham, Amund Maage, Marian K Malde

**Affiliations:** 1 National Institute of Nutrition and Seafood Research (NIFES), P.O. Box 2029 Nordnes, N-5817 Bergen, Norway; 2 Department of Science and Technical Education (DOSATE), Makerere University, P.O. Box 7062, Kampala, Uganda

**Keywords:** Zinc, iron, Uganda, Kumi District, micronutrients, 24-hour recall

## Abstract

**Background:**

Iron and zinc are essential micronutrients for humans and deficiency of the two elements is widespread in the world with the highest prevalence in less developed countries. There are few data on dietary intake of iron and zinc in Uganda, and no food composition table is available. There is hardly any widely published literature that clearly documents the quality of Ugandan children's diet. Thus information of both food intake and the concentration of these trace elements in local food ingredients are needed in order to assess daily intake.

**Methods:**

The present study focused on the iron and zinc content in selected foods and intake of the micronutrients iron and zinc among schoolchildren in Kumi District, Uganda. Over a period of 4 weeks single 24-hour dietary recall interviews were carried out on a convenience sample of 178 schoolchildren (9-15 years old). Data from the dietary recalls was used when selecting foods for chemical analysis.

**Results:**

Results from this study showed that the iron concentrations varied, and were high in some cereals and vegetables. The zinc concentrations in foods generally corresponded with results from other African countries (Mali and Kenya). Data from the 24-hour dietary recall showed that the daily Recommended Nutrient Intake (RNI) was met for iron but not for zinc.

**Conclusions:**

The schoolchildren of Kumi district had a predominantly vegetable based diet. Foods of animal origin were consumed occasionally. The iron content in the selected foods was high and variable, and higher than in similar ingredients from Kenya and Mali, while the zinc concentrations were generally in accordance with reported values. The total daily zinc (mg) intake does not meet the daily RNI. The iron intake is adequate according to RNI, but due to iron contamination and reduced bioavailability, RNI may not be met in a vegetable based diet. More studies are needed to investigate possible sources of contamination.

## Background

In low-income countries it has been estimated that about 12 million children below the age of five years die annually due to infection and malnutrition, with malnutrition contributing to half of the mortality [1]. Nutritional deficiency is one kind of malnutrition. A staggering number of children are affected by micronutrient deficiencies which are of critical concern in developing countries [2]. In the early 1990's the problem of micronutrient deficiencies, referred to as the "hidden hunger", was given worldwide attention [3]. It became apparent that large parts of the developing world suffered from micronutrient malnutrition [4]. Iron deficiency is the most prevalent micronutrient deficiency in the world and 3 billion people worldwide are affected [5]. Iron deficiency exists in all countries, but the prevalence is highest in South East Asia (57%) and Africa (46%) [6]. The most vulnerable groups are pregnant women, children and adolescents due to the increased iron needs during pregnancy and also during rapid growth in children and adolescents [7]. In 2002, zinc deficiencies was included as a major risk factor in the global burden of disease, and in 2004 WHO/UNICEF included zinc supplements in the treatment of acute diarrhoea [8]. Despite this recognition, and that correction of zinc deficiency might have a great impact on the health situations in large populations of the developing world; zinc is still not included in the UN (1999) micronutrient priority list [9,10]. Milder and less severe zinc deficiency has been discovered in otherwise healthy infants and children, in both industrialized and developing countries [11]. The milder zinc deficiency can lead to growth retardation and is much more widespread than the severe version [12]. In some regions, among vulnerable groups such as growing children in populations subsisting on plant-based diets, milder or less severe zinc deficiency might be endemic [13]. Worldwide, the prevalence of zinc deficiency has been estimated to be almost 20% [14].

Although Uganda produces adequate amounts of food for its population, imbalances occur, that lead to food shortages both at a local and a regional level. The nutritional status in Uganda is generally poor, and under-nourishment is considered as one of the major health problems. Among children under five years old, it has been estimated that 40% of deaths are attributed to malnutrition, with 38% and 16% levels of stunting and underweight, respectively [15,16]. The prevalence of malnutrition in Uganda however, has mainly been described using anthropometric parameters and little is known about micronutrient status especially among schoolchildren. Studies have reported that pregnant women and children in Uganda are at high risk of iron deficiency [17,18], as a result of low levels of iron in their diets and limited iron supplementation, probably due to associated high costs [16]. The National Food and Nutrition Policy of Uganda focuses among other things on elimination of micronutrient disorders [15]. The micronutrients in focus are iodine, vitamin A and iron. There has been less focus on zinc both globally and in Uganda, and no national statistics on zinc deficiency is available. However, studies of nutritional adequacy of traditional foods in Uganda report that zinc content is low [19].

Dietary intake of children is influenced by many factors, including the available food in the household, time and resources allocated to child care, and the feeding practices [20]. There are to our knowledge no literature that clearly documents the quality of Ugandan children's diet and there is no national food composition table either. Given the high prevalence of malnutrition generally, more studies are needed to investigate the micronutrient content of foods consumed by Ugandan children.

Information on nutrient composition of food is useful and necessary for nutritional assessment, planning and implementation of food guidelines, nutrition education programmes and in research [21]. Many developing countries are still lacking satisfactory national food composition tables. However, a food composition table for Africa and some African countries have been established. At the African Food and Nutrition Congress in Harare, Zimbabwe in 1988, an African Network of Food Data Systems (AFROFOODS) was established to update the food composition data of the African countries [21]. East Central African Network of Food Data Systems (ECAFOODS) is the regional group for east African countries which includes Uganda. The group is working on updating and establishing food composition tables in this region. These regional food composition data bases are especially important for countries with lack of resources to establish a national food compositional table, but share a similar food supply to the countries in the region [22]. There are still factors that vary greatly between countries and ideally each country should have a national food composition data base.

Based on these facts the presented study was set up to study the iron and zinc content of the key food items in the diet of primary schoolchildren in Kumi district of Uganda. The present study was a part of a P.hd-project relating the health and nutritional status of the school children of Kumi district to learning achievement [23].

## Methods

### Ethical consideration

Ethical approval for this study was obtained from the District Officer of Security, the District Officer of Education and the head teachers of the respective schools. The study was a part of the project "Nutrition, Health and Learning Achievement: A Case of Primary School Children in Kumi District" [23], which was cleared by the Minister of Education, the national council for science and technology and the ethical committee for human studies. Consent was obtained from the parents/caretakers. In addition, informed consent was obtained orally from the students before the 24-hour recall interviews.

### Study area

The field work was carried out in Kumi District in eastern Uganda in the period 18.07.06 - 13.08.06. The district covers a total area of approximately 2821 km^2^, comprising three administrative counties; Bukedea, Kumi and Ngora (at the time). The climate in the district is equatorial with a bimodal type of rainfall received in the months of April-May and July-August, increasing southwards. There is a main dry season from December to February. The vegetation is predominantly savannah, although it is also punctuated by thickets, some forest plantations and riparian vegetation [24]. The district borders to the Lake Kyoga basin that consists of several lakes. Five of the lakes are present in Kumi district. Lake Kyoga basin is located in the south-west and the north of the district and is formed by the Victoria Nile that flows through the district. The wetland which covers 35% of the total area is very important to the livelihood of the people in the district [25]. In the Population and housing census from 1991 the population of Kumi district was about 237,000 of which 95% lived in the rural areas. The settlement patterns in the district reflect the resources available, a phenomenon in most districts of Uganda where agriculture is the major backbone of the district economy. The majority of the houses in the rural areas are huts, most of which are temporary, whereas in the urban areas it is more common to live in detached or semi-detached houses. In towns, the houses are inadequate and there is lack of maintenance for the few detached houses that are present [24].

### Subjects

The subjects in the present study were already enrolled in the main project. The sample size for the main project was determined using WHO cluster sampling procedure [22]. For the present study, purposive sampling was used, where three well performing and three poor performing schools were selected from the schools involved in the main project. The present study included a subsample of 180 children, 90 children from three well performing schools and 90 children from three poorly performing schools attending 4th and 5th grade in primary school. A sub-sample was selected taking practical reasons as transport and limited time period into consideration. The schoolchildren taking part in this research were 9-15 years old. The choice for the grades 4 and 5 was based on literature that fourth/fifth grade is a particularly susceptible time for learners, where they are transitioning into more complex cognitive mechanisms that can challenge their simple and sure knowledge base [26,27]. The pattern of declining scores from third to fourth grade has been previously reported [28]. All children sampled were from day schools.

One academically well and one academically poor performing school were selected from each county (based on academic performance of public examination results for the graduating schoolchildren of grade 7, for the previous year). For logistical and practical reasons classrooms in the selected primary schools were chosen as location of the dietary assessment.

### 24-hour dietary recall

The nutritional assessment was performed using the 24-hour recall method [29]. The single 24-hour recall method was selected as the most appropriate method of assessing the average intake of zinc and iron in the sample groups due to the low cost and less time consuming compared to other dietary assessments. A standard sample data sheet modified from Gibson [29] was used in the interviews. A pre-test of the interview was carried out before the actual interviews started. The interviews were carried out by one assistant and one teacher at each school (both natives and spoke the local language of the area; Ateso). The teachers were trained in English on how to carry out a 24-hour recall interview, before carrying out the interviews in the local language, Ateso. The local language was chosen to make it easier for the schoolchildren to freely express themselves and to feel comfortable. The training included questioning techniques, details required, do's and don'ts in case of failure of recall. The schoolchildren were permitted to mention foods in any order. Altogether 178 out of 180 interviews were completed (Table 1).

**Table 1:** Number of participants in the respective counties

County	Kumi		Ngora		Bukedea	
**School**	Kumi Township	Bazaar P/S	Ngora Township	Muru-Ikara P/S	Bukedea Township	Bukedea dem. P/S
**Schoolchildren** **(n)**	30	30	29	30	31	28

### Food sampling

The data on food intake collected in the 24-hour recall interviews was used as a basis to select foods for iron and zinc analyses. Both dry foods and fresh foods that were possible to sun-dry locally were included. This selection excluded certain foods such as tomatoes, cucumber and cabbage because of lack of facilities for drying or freezing the samples. The samples were obtained from the local market in each of the three counties Kumi, Bukedea and Ngora. The food sampling was undertaken in three days, one day at the market in each county. Each county has a fixed day in the week when the market takes place. From each market the vegetable and animal products given in the 24-hour recall were collected. The industrially processed food was only collected in one of the markets as it is assumed that these would not significantly differ between the three counties since they were distributed by the same company. The samples were collected according to the guidelines by International Network on Food Data Systems (INFOODS) for describing foods [30]. The fish bought at the market was sun dried or smoked at site of natives living in the area. The fish originated from the Lake Kioga basin (two different sites; Agwara and Namasale) and Lake Bisina. Meat samples from beef and goat were bought fresh at the market, and smoked in private households in Kumi and Bukedea. Fresh vegetables were sun dried on plastic sheets on the ground. Most samples were fully sundried, but due to cloudy weather some samples were partly dried inside during night. The drying time was between 2-3 hours when fully sundried and between 20-25 hours when partly sundried. A gate separated the un-tarred road from the site of sun-drying. Approximately 10 g of each sample were put in closed plastic bags (15 cm • 10 cm), and were transported to NIFES, Norway for analysis.

### Chemical analysis

The samples were stored at -20°C at the laboratory pending further processing. The sun-dried and smoked samples were homogenized when frozen. A mill (Princess 2194) was used to ground the vegetables. The frozen samples of fish, goat and cows' meat were already sun dried and smoked, which made it hard to separate the edible parts from the bones. Therefore these samples were boiled in 1.6 l NANO pure water for 30 minutes. Samples from the extraction were collected to detect any zinc or iron lost in the process of boiling. A more powerful mill (K25 DITO Electrolux) was used for homogenizing animal samples; fish, goat meat and cows' meat after boiling. Some samples required further homogenization because of the hard structure of the sample material. In these cases a mortar was used. The samples were stored in air tight plastic beakers (nunc beakers) at -20°C pending analyses. The frozen samples were freeze dried for 48 hours (Hetosicc model CD 52). The samples were weighed a second time and dry matter was calculated (Table 2). In the process of freeze-drying, the sample of baking flour boiled and polluted the other samples present in the freeze dryer at the same time by covering the samples with a thin white layer. Because of this, the procedure was changed for the remaining samples, determining the residue of evaporation using an oven (Termaks TS 5000). The samples were dried for 18 hours and cooled down in an exicator for 30 minutes before weighed a second time.

**Table 2:** Dry matter (%) of food samples collected in the respective counties

Sample		County	
	
	Kumi	Ngora	Bukedea
Beef	39	39	30
Goat	45	37	29
Irish potatoe	24	29	45
Sweet potatoe, betty	24	35	48
Sweet potatoe, kampala	n.s.	31	n.s.
Onion	18	46	8
Garlic	100	93	93
Eggplant	8	5	n.s.
Amaranthus p	18	22	n.s.
Amaranthus g	15	26	n.s.
Eboo	14	23	17
Echadoi	13	22	n.s.
Alilot	20	23	n.s.
Emoros	14	n.s.	n.s.

n.s. = No sample was collected.

The vegetable, oil and animal samples were all dissoluted using the standard method of microwave digestion before analyses [31]. Determination of the zinc and iron concentrations in the samples was performed using flame atomic absorption spectroscopy (Perkin Elmer model A-3300) at NIFES. The lamps used were hollow cathode lamps (HCL) from Perkin Elmer Co. The instrumental settings are given in table 3. The quantities of zinc and iron were calculated using a standard curve. The accuracy of the analytical methods of zinc and iron was assured by including the certified standard reference material (SRM) Oysters Tissue 1566b (Zn: 1424 ± 46 mg/kg, Fe:205.8 ± 6.8 mg/kg) (National Institute of Standards & Technology, USA), Cod Muscle 422 (Zn:19.6 ± 0.5 mg/kg, Fe 5.46 ± 0.30 mg/kg) (Community Bureau of References, Commission of the European communities), Tomato Leaves 1573 (Zn: 62 ± 6 mg/kg, Fe: 690 ± 25 mg/kg) and Wheat Flour 1567a (Zn:11.6 ± 0.4 mg/kg, Fe:14.1 ± 0.5 mg/kg) (National Bureau of Standards, USA). Limit of quantification for iron is 3 mg/kg in dry material and for zinc 1.8 mg/kg in dry material. The elemental analyses are all accredited by the Norwegian Metrology and Accreditation Service. The laboratories at NIFES are frequently participating in proficiency tests. The z-score is an independent assessment of a laboratory's competence, and z-scores within ± 2 are considered acceptable. All results obtained for the analytes presented in the present study showed z-scores within ± 2.

**Table 3:** The instrumental settings of FAAS when analysing for zinc and iron

Element	Wavelength	Slit of the monochromator	Nebulizer
	(nm)	(nm)	
Zn	213.9	0.7	Standard
Fe	248.3	0.2	Standard/High sensitive

### Statistical analysis

Data from 24-hour dietary recall interviews were plotted into spreadsheets of Microsoft Excel 2002, and this was also used to group and to present the data graphically.

## Results

### 24-hours dietary recall

**Figure 1:**
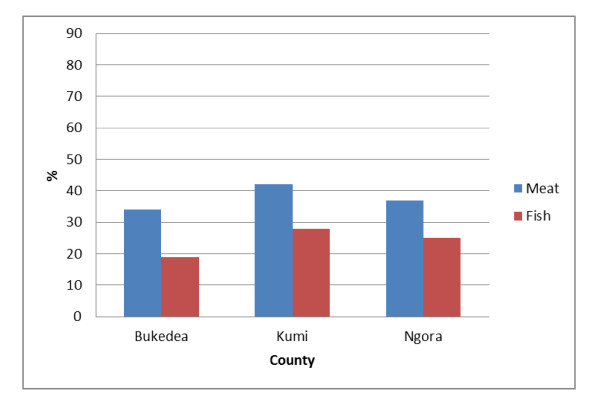
**Percentage of children that had eaten either meat or fish reported in 24-hour dietary recall by 9 to 15-years-old schoolchildren from Bukedea (n = 59), Kumi (n = 60) and Ngora (n = 59)**. The intake is illustrated as percentage of schoolchildren in each county eating fish and meat.

**Figure 2:**
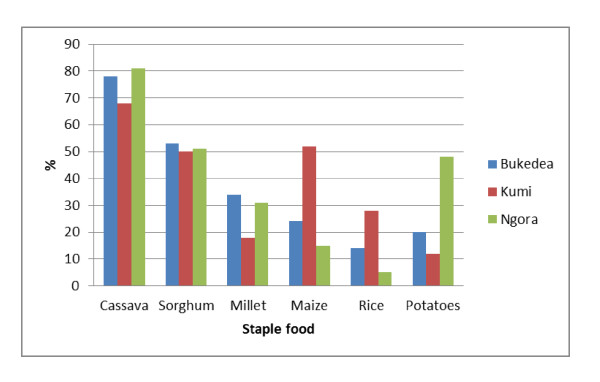
**Intake of staple foods reported in 24-hour dietary recall by 9 to 15-years-old schoolchildren Bukedea (n = 59), Kumi (n = 60) and Ngora (n = 59)**. The percentage of schoolchildren with intake of staple foods in each county is presented.

The schoolchildren of these communities consumed between two and three meals per day consisting of breakfast, lunch and supper, where breakfast was the most common meal to skip. The breakfast consisted most often of plain tea or tea with milk and ground nuts. Some children also got millet porridge, leftovers of rice or posho or maize meal (a stiff porridge made of maize flour), or wheat buns next to the tea for breakfast. A common lunch for the children eating at home included bread made out of cassava with sorghum or millet, and posho for some. The most common sauce was prepared from legumes, onions, cabbages and tomatoes. Fish and meat was included occasionally in the meals. In the evening before supper, tea or millet porridge with a side plate of ground nuts was often prepared. For supper, the main meal of the day, was often comprised of the same foods that were prepared for lunch. The intake of meat and fish reported in 24-hour recall interviews from the three counties is presented in Figure 1. In all counties a 10-15% higher intake of meat was reported than fish (Bukedea 15%, Kumi 14%, and Ngora 12%). There was also a difference in intake between the counties. The meat and fish intake were highest in Kumi followed by Ngora and Bukedea. The intake of staple foods reported in 24-hour recall is presented in Figure 2. The three staples (cassava, sorghum and millet used in bread baking) were the most frequently reported. Intake of cassava was reported by 70-80% of the schoolchildren, sorghum about 50%, and millet about 20-30% in all three counties. The intake of maize flour, which is used in making a stiff porridge (posho), varied more between counties. Intake of maize was reported by 52%, 24% and 15% in Kumi, Bukedea and Ngora, respectively. Rice and potatoes were on the contrary less frequently reported.

### Iron and zinc content of food samples

The iron content of fish and meat from Kumi, Ngora and Bukedea are presented in Table 4. The results showed that all samples contained detectable amounts of iron, and that iron content varied between samples and even those of the same species (e.g. fish). The iron content in samples of beef and goat meat was higher for Bukedea than for Kumi and Ngora. Samples of whole fish had also a higher content of zinc than samples of fillet independent of species. The fillets of Nile Perch and Tilapia, bought from Bukedea and Kumi markets, had zinc concentrations varying from 5-10 mg/kg. Whole samples of the same species collected from Ngora market had zinc concentrations of 50 mg/kg. The zinc content in the meat samples did not vary between counties in the same pattern as the iron concentrations. The samples of goat meat and beef had a concentration between 13 mg/kg and 20 mg/kg except for the sample of beef collected in Kumi that had a zinc concentration of 6 mg/kg (Table 4). The dry matter percentage for the samples of meat and fish is given in Table 2. The concentration of iron and zinc in the extracted water samples from boiled meat and fish were all under the limit of quantification when analysed at FAAS.

**Table 4:** Iron and zinc content (mg/kg) in fish fillet and meat samples collected in the respective counties

Sample	Species	Scientific name		Iron (mg/kg)			Zinc (mg/kg)	
			
			Kumi	Ngora	Bukedea	Kumi	Ngora	Bukedea
Fish	Omena	*Rastrineobola argentea*	120^a^	1000^a^	110^a^	220^a^	200^a^	220^a^
	Ioyo	n.n.	130^a, c^	160^a^	*	70^a^	120^a^	170^a^
	Nile Perch	*Lates niloticus*	6^b, d^	43^a, d^	10^b, d^	5^b, c^	50^a, c^	10^b, c^
	Tilapia	*Oreochromis variabilis*	3.3^b, d^	24^a, d^	9^b, c, d^	6^b, c^	50^a, c^	8^b, c^
	Catfish	*Siluriformes*	28^a^	n.s.	n.s.	40^a, c^	n.s.	n.s.
	Lungfish	*Protopterus aethiopicus*	7^b, c^	n.s.	n.s.	37^b, c^	n.s.	n.s.
Meat	Beef	*Bos indicus*	16	13	120	6	19	17
	Goat	*Hemitragus*	18	14	100	14	14	20

The values refer to sun dried fish samples and wet weight meat samples. Results are given as mean of two parallel dissolutions.

^a ^Sample of whole fish.

^b ^Sample of fish fillet.

^c ^Values with Relative Standard Deviation (RSD) between 10% and 20%.

^d ^The fish samples were smoked.

*The sample is rejected because of unacceptable high variation.

n.s. = No sample was collected.

The iron and zinc content in cereals is presented in Table 5. We observed a variation in iron content in the different types of flours and even in the flours bought at the same market. The iron content was highest in sorghum flour collected from Ngora market (7000 mg/kg) and lowest in rice collected from Bukedea market (3.5 mg/kg). The highest zinc concentration was found in millet flour in the sample collected at Kumi market (24 mg/kg). Maize flour collected from Ngora market had the lowest zinc concentration (3.6 mg/kg). The results showed that the zinc content was about 3 times higher in sorghum and millet than in maize and cassava flours.

**Table 5:** The iron and zinc content (mg/kg wet weight) of cereal samples collected in the respective counties

Sample	Scientific name		Iron (mg/kg)			Zinc (mg/kg)	
		
		Kumi	Ngora	Bukedea	Kumi	Ngora	Bukedea
Cassava flour	*Manihot esculenta *Crantz	80	n.s.	*	6	n.s.	5
Sorghum flour	*Sorghum bicolor *(L.) Moench.	600	7000^a, b^	90	15	18	16
Millet flour	*Eleusine coracana *Gaertn.	570^a, b^	*	70	24	17	20
Maize flour	*Zea mays L*.	30	53	70	4.2	3.6	7
Wheat flour	*Triticum aestivum *L.	13	n.s.	n.s.	8	n.s.	n.s.
Rice	*Oryza sativa *L.	9	9	3.5	14	14	14
Sesame seeds	*Sesamum orientale *L.	500^c^	n.s.	n.s.	44	n.s.	n.s.

The results are given as a mean of two parallel dissolutions.

^a ^Mean value of four parallel dissolutions.

^b ^Values with Relative Standard Deviation (RSD) between 10% and 20%

^c ^Values with Relative Standard Deviation (RSD) between 20% and 30%

*The sample is rejected because of unacceptable variation.

n.s. = No sample was collected.

The concentrations of iron and zinc in starchy roots are presented in Table 6. The results show that the iron concentrations were higher than zinc concentrations in all samples. The highest iron content was found in Irish potato (140-500 mg/kg) and lowest in sweet potato (42-46 mg/kg). The zinc concentration was also higher in Irish potatoes than sweet potatoes (3.3-3.5 mg/kg in Irish potatoes and 1.6-3.0 mg/kg in sweet potatoes). The iron concentration in nuts and legumes collected from Kumi district is presented in Table 7. The results show that beans had higher iron content than ground nuts (64-180 mg/kg and 20-34 mg/kg respectively). The lowest iron concentration recorded for ground nuts was 20 mg/kg in Serenut collected from Bukedea market and the highest was 34 mg/kg in Obino collected from Kumi market. Among the beans, iron concentration varied from 64 mg/kg to 90 mg/kg with an exception of soya beans sampled from Bukedea that contained 180 mg/kg. In the group of peas the highest value analysed was 1300 mg/kg in green grams collected from Bukedea market and the lowest (42 mg/kg) in green grams collected from Kumi market. The zinc content in beans and ground nuts were similar, (22-52 mg/kg and 24-30 mg/kg respectively). Among the analysed beans, soya beans had the highest zinc concentrations (34-52 mg/kg).

**Table 6:** Iron and zinc content (mg/kg wet weight) in starchy roots collected in respective counties

Sample	Scientific name		Iron (mg/kg)			Zinc (mg/kg)	
		
		Kumi	Ngora	Bukedea	Kumi	Ngora	Bukedea
Cassava dry	*Manihot esculenta *Crantz	n.s.	n.s.	50	n.s.	n.s.	7
Irish potato	*Solanum tuberosum *L	140	400^a, c^	500^c^	3.3	3.5	3.5^b^
Sweet potato, Betty	*Ipomoea batatas *(L.) Lam	*	42	56	3	1.6	1.7
Sweet potato Kampala	*Ipomoea batatas *(L.) Lam	n.s.	43	n.s.	n.s.	2.3^a^	n.s.

For potatoes the values are for non-peeled samples. Results are given as a mean of two parallel dissolutions.

^a ^Mean value of four parallel dissolutions.

^b ^Values with Relative Standard Deviation (RSD) between 10-20%

^c ^Values with Relative Standard Deviation (RSD) between 20-30%

*The sample was rejected because of unacceptable variation.

n.s. = No sample collected.

**Table 7:** The iron and zinc content (mg/kg wet weight) in nuts and legumes collected in Kumi, Ngora and Bukedea

Sample	Species	Scientific name		Iron (mg/kg)			Zinc (mg/kg)	
		
			Kumi	Ngora	Bukedea	Kumi	Ngora	Bukedea
Ground nuts	Serenut	*Arachis hypogaea *L.	25	n.s.	20	30	n.s.	26
	Obino	*Arachis hypogaea *L.	34	n.s.	n.s.	25	n.s.	n.s.
	Otira	*Arachis hypogaea *L.	20	n.s.	n.s.	30	n.s.	n.s.
	Valencia	*Arachis hypogaea *L.	n.s.	n.s.	26	n.s.	n.s.	27
	Agolitom	*Arachis hypogaea *L.	n.s.	23	n.s.	n.s.	24	n.s.
Beans	Soya	*Glycine max*	80	70	180	52	38	34
	Kaneybwa	*Phaseolus vulgaris *L	70	70	54	26	24	24
	TZ	*Phaseolus vulgaris *L	n.s.	70	90	n.s.	22	27
	Saitot	*Phaseolus vulgaris *L	64	n.s.	n.s.	33	n.s.	n.s.
	White	*Phaseolus vulgaris *L.	70	n.s.	n.s.	34	n.s.	n.s.
	Yellow	*Phaseolus vulgaris *L.	70	n.s.	n.s.	34	n.s.	n.s
Peas	Green grams	*Phaseolus aureus *Roxb.	42	900	1300	27	31	27
	Cow peas	*Vigna anguiculata *Crantz	61	45	45	32	31	32

The results are given as a mean of two parallel dissolutions.

n.s = No sample was collected.

The iron and zinc concentrations in vegetables and fruit sampled are presented in Table 8. The results showed that leafy vegetables had higher iron concentrations than bulky vegetables (32-340 mg/kg in leafy vegetables and 5.4-36 mg/kg in bulky vegetables). The zinc concentration did not vary to the same extent as the iron concentration iron between bulky and leafy vegetables (1.1-7.3 mg/kg in bulky vegetables and 3.2-7.0 mg/kg in leafy vegetables). The highest zinc content was found in garlic sampled in Bukedea (3.7 mg/kg) and the lowest zinc content was found in onion and eggplant collected at Ngora market (1.1 mg/kg in both).

**Table 8:** The iron and zinc content (mg/kg wet weight) in vegetables and fruit collected in respective counties

Sample	Species	Scientific name		Iron (mg/kg)			Zinc (mg/kg)	
			
			Kumi	Ngora	Bukedea	Kumi	Ngora	Bukedea
Bulky	Onion	*Allium cepa *L.	6	35	36	1.9	1.1	3.7
	Garlic	*Allium sativum *L.	6.5	12	12	9	7	7.3
	Eggplant	*Solatum Melonga *L	8	5.4^a^	n.s.	2.3	1.1	n.s.
Leafy	Amaranthus, purple	*Amaranthus cruentus *L	150	340	n.s.	8	4	n.s.
	Amaranthus, green	*Amaranthus hybridus *L	130^a^	180	n.s.	7	3.2	n.s.
	Eboo	*Vigna anguiculata Crantz*	32	80	160	5	5	7
	Echadoi	*Cleome gynandra *L	160	100	n.s.	6.3	6	n.s.
	Alilot	*Hibiscus esculentus *L	140	320	n.s.	6	5.3	n.s.
	Emoros	*Cyphostemma adenocaule*	140	n.s.	n.s.	3.4	n.s.	n.s.
Fruit	Tamarines	*Tamarindus indica *L	n.s.	n.s.	56	n.s.	n.s.	11

The results are given as a mean of two parallel dissolutions.

n.s. = No sample was collected.

^a ^Values with Relative Standard Deviation (RSD) between 10-

The content of zinc and iron in spices, sugar and baking powder are presented in Table 9. Samples of spices were limited and sugar was the only sample in this category. Of the four types of curry sampled; Munno, Tayara and Turkey had a higher iron concentration than Royco. The same pattern was seen in zinc concentrations. The four collected samples of vegetable oil had all iron content under the limit of quantification when analysed at FAAS.

**Table 9:** Iron content (mg/kg wet weight) in spices, sugar and baking powder collected in respective counties

Sample	Species	Scientific names		Iron (mg/kg)			Zinc (mg/kg)	
		
			Kumi	Ngora	Bukedea	Kumi	Ngora	Bukedea
Sugar		*Saccharum officinarum *L.	16	36	38	< 1.8*	< 1.8*	2
Salt		Sodium chloride	33	n.s	22	2.4	n.s.	< 1.8*
Curry	Munno	*Curcama longa L*.	1200	n.s.	n.s.	30	n.s.	n.s.
	Royco	*Curcama longa L*.	62	n.s.	n.s.	5	n.s.	n.s.
	Tayara	*Curcama longa L*.	1000	n.s.	n.s.	70	n.s.	n.s.
	Turkey	*Curcama longa L*.	n.s.	n.s.	800	n.s.	n.s.	60
Pilao		*Sysygium aromanticum *L, Cuminum cyminum L., Elettaria cardamomum Maton	160	n.s.	n.s.	24	n.s.	n.s.
Baking powder		Sodium bicarbonate	10	n.s.	n.s.	< 1.8*	n.s	n.s.

The results are given as a mean of two parallel dissolutions.

n.s. No sample was collected.

* Limit of quantification = 1.8 mg/kg dry weight.

### Estimation of iron and zinc intake

In addition to values from the zinc and iron analysis information from the Food Composition Table for Mali (Table de composition d'aliments du Mali (TACAM) [32] was used to estimate the total daily intake of iron and zinc from common Ugandan meals (Table 10), some of whose meals are similar to the Ugandan meals. Proportions and weights of ingredients were used for estimating portion size for lunch and supper. The sauce included ingredients that were not sampled in this study (tomatoes and cabbage); hence values of zinc and iron from TACAM were used. An example of a schoolchild's diet of one day is: millet porridge for breakfast (400 g), lunch (400 g) and supper (400 g) made from the staples cassava and sorghum or millet, and sauce made of legumes, onion, cabbage and tomatoes. A snack of ground nuts (100 g) during the day was common in this season. The Malian dish Guenzinkini is similar to a common Ugandan dish which contains a maize, sorghum or millet as staple and a sauce named Tigadeguenan made of ground nut paste, cabbage, onion, tomatoes and water. The dish contains 70% staple and 30% sauce, which means that a dish of 400 g contains 280 g staple and 120 g sauce [32]. This common diet, reflecting the daily intake of a schoolchild in Kumi district, has an estimated energy intake of ≈7330 kJ (1750 kcal).

**Table 10:** An example of the estimated content of meals^f ^eaten by Ugandan schoolchildren, and the total intake of iron (mg) and zinc (mg) from the respective meals

Meal	Dish/snack	Amount (g)	Iron (mg)^e^			Zinc (mg)
			
			Lowest	Mean	Highest	Mean
Breakfast	Millet porridge	400	3	14	23	0.8
Snack	Ground nut	100		2.5		2.7
Lunch/Supper	Staple 1^a^	280	3	10	23	0.3
	Staple 2^b^	280	2.3	3.9	5.3	0.3
	Sauce^d^	120		1.1^c^		0.1
Whole day	Including staple 1	1300	13	38	73	4.1
Whole day	Including staple 2	1300	11	25	37	4.2

^a ^Staple 1 = Cassava and sorghum

^b ^Staple 2 = Maize porridge

^c ^Iron concentration from Food Composition Table for Mali (Table de composition d'aliments du Mali (TACAM)) was used to calculate the content

^d^The sauce contains ground nut paste, tomatoes, cabbage and onion.

^e^Iron concentrations are calculated by using lowest, mean and highest concentration of the analysed samples.

^f^The dish contains 70% staple and 30% sauce, which means that a dish of 400 g contains 280 g staple and 120 g sauce [32]. This common diet, reflecting the daily intake of a schoolchild in Kumi district, has an estimated energy intake of ≈7330 kJ (1750 kcal). In the total intake of a whole day the dish of lunch and supper is included twice as this dish is considered to be eaten for both meals.

## Discussion

### 24-hour dietary recall

The relationship between the subject and interviewer (schoolchild and teacher) might have had an influence on the report of food intake. Any negative effect of these factors was minimized by giving detailed instructions to each interviewer, and by using standard data sheets when interviewing. During the survey the permanent assistant was updated as experience was gained. The day of the week when trade at the local market took place in the county, and also other special occasions can give a day-of-the-week effect on the data [33]. In this survey, these potential errors were reduced by carrying out 24-hour recall interviews at different weekdays, including weekends, as recommended in the literature [29]. Optimally the method should not only have counteracted for between person variability, but also for within person variability by carrying out a repeated 24-hour recall on a sub-sample [34]. In this survey, time limitations did not permit this, and more so, the study was intended to give a general overview of the children's intake rather than relating it to their nutritional status, which would require repeated measures. In general, the outcome of a 24-hour recall study, is depending on certain factors such as memory, ability to estimate portion sizes, degree of motivation and the persistence of the interviewer [35]. The schoolchildren taking part in this research were 9-15 years old. Some studies have shown that this age group is old enough to remember and estimate food quantities [36], while other studies indicate that children have problems in reporting food quantities [37]. Our experience was that children at this age were able to recall food items, but had problems with recalling and/or estimating exact amounts. Therefore, only food items were recorded. The compliance to the 24-hour recall is considered to be relatively high due to a small respondent burden [29]. This was also experienced in this survey. Due to the relatively high number of students interviewed, and a relatively low variety in food items available in the area, it is likely that the results give an acceptable overview of the staple food consumed by the children.

### Staple food

According to the 24-hour recall interviews, the diet among schoolchildren in Kumi district contained the staples; cassava, millet, sorghum, maize, rice, sweet potatoes and Irish potatoes. Cassava and sorghum were most frequently reported (Figure 2). Less than 42% of the children reported intake of meat and less than 28% reported intake of fish (Figure 1). Beef, goat meat, Nile perch and Tilapia were the most frequently reported foods of animal origin. Nuts and legumes were both common parts of the diet, especially in absence of fish and meat. Leafy vegetables were rarely reported as a part of the diet. There was no school feeding programs in the district at the time, which made the schoolchildren run home for lunch during the midday break. The children staying too far away remained at school generally without eating lunch (a common scenario in the developing world). Some children brought money and had the possibility to buy roasted maize, mandazi (made from wheat flour, oil and sugar) or bolingo (rice ball with curry powder) from local vendors during the midday break at some schools. During this fieldwork it was ground nut season in the area, and in the season it is common amongst the schoolchildren to pick and eat raw ground nuts.

Higher intakes of meat, fish and posho in Kumi county may be attributed to socio-economic status of the communities. Generally, Kumi was the headquarters for district administration, and there were therefore better standards of living here compared to the other two counties.

### Iron and zinc content of selected food items

The iron and zinc content in the vegetable based samples did not seem to vary between the counties. However, the meat samples had a higher concentration of iron in Bukedea compared to the two other counties. This difference might be due to differences in the process of smoking, as the samples from Bukedea were smoked in a different household of practical reasons. The iron and zinc content varied between samples and even in those of the same species (e.g. fish). Samples of whole-fish had higher content of iron than the samples of fillet as expected since whole fish samples also contains the liver, where fish store iron. Comparing the iron concentrations in the food items reported in this study with other countries, iron concentrations in this study were high (Table 11). High iron levels in cereals reported in this study might be due to contamination of iron from equipment used in the grinding process [38]. In a study of iron concentrations in fish meal, it was found that the fish meals were contaminated from the equipment used in the process. During the first hours of production the iron concentration of the meal increased to a level of 800 mg/kg, and further decreased with time [39]. The iron concentration of the fishmeal would therefore vary with the time in production. Further, in a study from Tanzania which also reported high iron levels in cereals and vegetables at the same time as it was found extremely low bioavailability of iron. The explanation of the high iron concentrations in this study may be that the samples were contaminated [40] from soil. Soil residues and dust can settle on the surface of cereals and vegetables during air drying [41]. Hence, the high concentrations of iron in the leafy vegetables (Table 8) in this study might be due to soil contamination from dust at the market or in the process of drying. The high iron concentration in potatoes might also be explained by soil and earth remnants, as the analysed samples were unpeeled (Table 6). Contrary to the iron levels, the results showed that the zinc concentration in neither leafy vegetables nor the potatoes had especially high concentrations. Hooda et al. [42] analysed Ugandan soil for iron and zinc in a study of geophagy (soil consumption) and nutrient bioavailability of minerals. The iron and zinc concentrations were 14825 mg/kg and 25 mg/kg, respectively. The high iron concentrations and the large difference between iron and zinc concentrations support the explanation that some food samples in our study might have got extra iron from the soil. In the same study it was concluded that soil used for consumption potentially can reduce the absorption of iron and zinc and contradict that geophagic materials can be a source of nutrient supplementation. Another study of geophagy and the soil in Uganda report iron levels in seven soil samples in the range of 16643 mg/kg to 81696 mg/kg [43]. The study concludes that in consumption of 5 g soil between 0.37 mg and 4.8 mg iron are available for absorption, but the bioavailability of contamination iron is still of debate [41,42]. In a Tanzanian study of iron availability, it was also observed that the iron content of cereals and vegetables was higher than values in the African Food Composition Table while the iron values of legumes agreed. The zinc concentrations in this study fall within the range of the studies of Kenya and Mali for cereals, legumes and fish (Table 10). The zinc concentrations of meat appear to be slightly below the values given from Kenya and Mali.

**Table 11:** Comparison of iron and zinc levels (mg/kg) in some Ugandan foods with iron and zinc levels in similar foods from Kenya and Mali

Food		This study*		Kenya^a^		Mali^b^	
		**Fe**	**Zn**	**Fe**	**Zn**	**Fe**	**Zn**
Cereal	Sorghum	19-7000	15-18	41	16	58	21
	Millet	70-570	17-24	27	12	58	29
	Maize	30-70	3.6-7	35	18	11	-
Legumes	Ground nuts	20-34	24-30	46	33	39	-
	Soy beans	70-180	34-52	64	23	61	-
	Brown beans	54-90	22-33	75	28	-	-
Vegetables	Irish potatoe	140-500	3.3-3.5	-	-	11	-
	Amaranthus	130-340	3.2-8	-	-	89	-
Fish	Nile Perch	6-10	5-10	9	-	112	-
	Tilapia	3.3-9	6-8	9	-	-	-
Meat	Beef	13-120	6-19	12	30	46	23
	Goat meat	16-120	14-20	12	29	23	40

^a ^WorldFood Dietary Assessment System 2.0 [46].

^b ^Food Composition Table for Mali [32].

*Values for this study are presented with mean and range in parenthesis.

### Iron and zinc intake

The estimated energy intake of 1750 kcal/day (Table 10) is lower than the recommended daily energy requirements for children at the age of 9-15 years (boys: 1978-2990 kcal/d and girls: 1854-2449 kcal/d) [44]. When estimating the iron and zinc content of the dish, iron concentration was calculated using three values of; lowest, highest and mean concentration. This was done to illustrate the different outcome when samples of the same food were included in the meals. The zinc content of the meals was calculated using only mean values of the ingredients as less variation between the samples occurred. Examples including two of the most common staples in the district are given; cassava mixed with sorghum and a stiff porridge (posho) made of maize flour (Table 11). Cassava, which was most frequently reported in Kumi district, was substituted with millet, which was included in TACAM. The substitution was done, assuming that the same amount of cassava and millet was used in the dish. As in the description of a common Ugandan diet, it is assumed that the same dish was served for lunch and supper; hence the values given for the whole day include both meals. In this example all values except for iron and zinc concentrations of the sauce are from this study. The zinc and iron concentrations in the sauce are from TACAM. The total intake of iron and zinc in one day was in this example higher when including the staple of cassava and sorghum in the main meals compared to maize flour. The total daily intake of iron, when the staple of cassava and sorghum are included, was estimated to be 13 mg, 38 mg, 73 mg at lowest, mean and highest concentrations, respectively. The mean zinc intake in this example is 4.1 mg/day. If maize porridge is included in the main meals, lunch and supper, the total intake of iron is 11 mg, 25 mg and 37 mg at low, mean and highest iron concentrations respectively, and the total daily intake of zinc is 4.2 mg. Out of the total daily intake of zinc, ground nut snack contribute with 65%. Thus, ground nuts are an important zinc source during the ground nut season (Table 11). In a western type of diet including vegetables, fruits, meat and fish, the bioavailability of iron is considered to be about 15% [38]. Due to the high content of phytic acid in the common foodstuffs, the bioavailability levels of 5% and 10% for iron are considered to be realistic in developing countries [38]. The diet described consists of cereals and legumes that are high in phytic acid which is considered as the main inhibitor of zinc and iron availability [45]. According to a similar study in Tanzania, a similar diet had an iron bioavailability of 5% [40]. The intake of iron from the estimated Ugandan diet is above the RNI for children, 7-10 years old, and adolescents 11-14 years old for dietary iron at 5% and 10% bioavailability in the diets of mean and high iron concentrations. The RNIs referred to are derived from the estimates of average individual dietary requirements [38].

## Conclusions

In this study it was found that schoolchildren of Kumi district had a predominantly vegetable based diet. Foods of animal origin were consumed occasionally. The iron content in the selected foods was high and variable, and some vegetables and cereal exceeded the iron concentrations in meats. The iron content in the food samples reported in the present study is higher than in similar ingredients from Kenya and Mali. The iron intake is adequate according to RNI, but due to the chemical form the iron may have low bioavailability.

The zinc concentrations are generally in accordance within reported values. Ground nuts are an important zinc source during the ground nut season. However, the total daily zinc (mg) intake does not meet the daily RNI. More studies are needed to investigate possible sources of iron contamination.

## Competing interests

The authors declare that they have no competing interests.

## Authors' contributions

IT-A participated in the design of the study, carried out the field work, interpreted the dietary recalls, analyzed the food samples, and drafted the first version of the manuscript. HA conceived of the PhD study which the present study is a part of, participated in design of the present study, and had the main responsibility of coordinating the fieldwork. AM participated in the design of the study, and in the interpretation of the chemical analysis. MKM conceived the study, participated in its design and coordination, and finalized the manuscript. All authors have read and approved the final manuscript.
